# Case Report: Flavoring-Related Lung Disease in a Coffee Roasting and Packaging Facility Worker With Unique Lung Histopathology Compared With Previously Described Cases of Obliterative Bronchiolitis

**DOI:** 10.3389/fpubh.2021.657987

**Published:** 2021-05-20

**Authors:** R. Reid Harvey, Brie H. Blackley, Eric J. Korbach, Ajay X. Rawal, Victor L. Roggli, Rachel L. Bailey, Jean M. Cox-Ganser, Kristin J. Cummings

**Affiliations:** ^1^Respiratory Health Division, National Institute for Occupational Safety and Health, Morgantown, WV, United States; ^2^HealthPartners, Saint Paul, MN, United States; ^3^Department of Pathology, Duke University, Durham, NC, United States

**Keywords:** diacetyl, 2, 3-pentanedione, coffee roasting and packaging, obliterative bronchiolitis, flavoring-related lung disease, case report

## Abstract

Occupational exposure to diacetyl, a butter flavor chemical, can result in obliterative bronchiolitis. Obliterative bronchiolitis is characterized by exertional dyspnea, fixed airflow obstruction, and histopathologic constrictive bronchiolitis, with bronchiolar wall fibrosis leading to luminal narrowing and obliteration. We describe a case of advanced lung disease with histopathology distinct from obliterative bronchiolitis in a 37-year-old male coffee worker following prolonged exposure to high levels of diacetyl and the related compound 2,3-pentanedione, who had no other medical, avocational, or occupational history that could account for his illness. He began working at a coffee facility in the flavoring room and grinding area in 2009. Four years later he moved to the packaging area but continued to flavor and grind coffee at least 1 full day per week. He reported chest tightness and mucous membrane irritation when working in the flavoring room and grinding area in 2010. Beginning in 2014, he developed dyspnea, intermittent cough, and a reduced sense of smell without a work-related pattern. In 2016, spirometry revealed a moderate mixed pattern that did not improve with bronchodilator. Thoracoscopic lung biopsy results demonstrated focal mild cellular bronchiolitis and pleuritis, and focal peribronchiolar giant cells/granulomas, but no evidence of constrictive bronchiolitis. Full-shift personal air-samples collected in the flavoring and grinding areas during 2016 measured diacetyl concentrations up to 84-fold higher than the recommended exposure limit. Medical evaluations indicate this worker developed work-related, airway-centric lung disease, most likely attributable to inhalational exposure to flavorings, with biopsy findings not usual for obliterative bronchiolitis. Clinicians should be aware that lung pathology could vary considerably in workers with suspected flavoring-related lung disease.

## Introduction

Flavoring-related lung disease was first described in 2000 when butter-flavored microwave popcorn production was associated with a cluster of clinical bronchiolitis obliterans, or obliterative bronchiolitis, in former workers (1, 2). Clinical bronchiolitis obliterans will refer to the clinical syndrome and obliterative bronchiolitis will refer to the lung pathology for the purposes of this report. Diacetyl (2,3-butanedione), an alpha-diketone, was determined to be the component of liquid butter flavoring responsible for disease. Unlike other known occupational causes of obliterative bronchiolitis, acute symptoms in these cases did not follow a recognized overwhelming exposure; rather, clinical progression was insidious, marked by exertional dyspnea and airflow obstruction that did not improve significantly following bronchodilation (fixed obstruction). The few cases that underwent biopsy had histopathologic findings consistent with obliterative bronchiolitis (2), typically characterized by concentric fibrosis and destruction of the bronchioles (3). Since the sentinel microwave popcorn plant was identified, additional cases of indolent onset clinical bronchiolitis obliterans have been diagnosed, often without biopsy, in workers in a number of other industries, including flavoring manufacturing, food (other than popcorn) production, and coffee roasting and packaging (4–7).

In 2016, we conducted a National Institute for Occupational Safety and Health (NIOSH) Health Hazard Evaluation that included medical and industrial hygiene surveys following a request from management at a coffee roasting and processing facility that added liquid flavorings to coffee. We describe a case of advanced lung disease in a former coffee worker following prolonged exposure to high levels of diacetyl and the related compound 2,3-pentanedione. The availability of lung tissue in this case provides an opportunity to re-examine the histopathologic consequences of flavoring exposure.

## Case Report

In 2009, A 37 year-old male former smoker (1 pack-year history) with no significant medical history began working in the flavoring room and grinding area of a coffee facility that had no respiratory protection requirements or recommendations. He reported mucous membrane irritation, as well as wheezing and chest tightness that worsened with exertion when working in the flavoring room and grinding area beginning in 2010 after several months of employment. Initially, these symptoms resolved after he left those areas of the facility, but his work-related symptoms slowly progressed until there was no discernible work-related pattern. In 2013, he moved to the packaging area but continued to flavor and grind coffee at least 1 full day per week. Beginning in 2014, he developed dyspnea, intermittent cough, and a reduced sense of smell that did not improve when away from work. He reported that during 2014–2016, he experienced frequent upper respiratory infections and was treated with antibiotics several times for presumed pneumonia but received no diagnostic testing.

Our 2016 evaluation of the workforce included pulmonary function testing (8). This worker's spirometry revealed a moderate mixed obstructive and restrictive pattern, and impulse oscillometry was consistent with peripheral airways obstruction; neither the spirometric nor oscillometry measures improved post-bronchodilator (Table 1). We recommended the patient seek care from a pulmonologist for evaluation of potential flavoring-related lung disease based on his symptoms, pulmonary function testing, and work history.

**Table 1:** Lung function testing results of worker diagnosed with flavoring-related lung disease, September 2016–July 2019.

	**Reference^€^(9)**	**August 2016**	**September 2016**	**October 2016**	**June 2017**	**October 2017**	**January 2018**	**July 2019**
**Spirometry**								
FVC^*^, Liters (% predicted)	4.01	3.22 (80%)	3.05 (74%)	3.28 (80%)	2.39 (58%)	2.60 (64%)	2.96 (73%)	2.68 (62%)
${\text{FEV}}_{1}^{\text{†}}$, Liters (% predicted)	3.18	2.07 (65%)	1.95 (59%)	1.98 (60%)	1.69 (52%)	1.78 (55%)	1.96 (61%)	1.89 (55%)
FEV_1_/FVC (%)	79%	64%	64%	60%	71%	68%	66%	71%
TLC^‡^ Liters (%)	5.24	–	–	5.36 (102%)	–	–	–	–
DL_CO_^£^ mL/mmHg/min (%)	27.3	–	–	24.0 (88%)	–	–	–	–
Impulse Oscillometry	Upper limit of normal (10)							
${\text{R}}_{5}^{**}$ (cm H_2_O/L/s)	3.96	4.59	–	–	–	–	–	–
${\text{R}}_{20}^{\text{†}†}$ (cm H_2_O/L/s)	3.20	3.26	–	–	–	–	–	–
Fres^‡‡^ (Hz)	12	19	–	–	–	–	–	–
AX^££^ (cm H_2_O/L/s)	3.60	12.34	–	–	–	–	–	–
R_5−20_^€€^ (cm H_2_O/L/s)	0.76	1.33	–	–	–	–	–	–

* *Forced vital capacity*;

† *Forced expiratory volume in one second*;

‡ *Total lung capacity*;

£ *Diffusing capacity of the lung for carbon monoxide*;

€ *Reference values for spirometry derived from National Health and Nutrition Examination Survey (NHANES) III*;

** *R5: resistance at 5Hz*;

†† *R20: resistance at 20Hz*;

‡‡ *Fres: resonant frequency*;

££ *AX: reactance area*;

€€ *R_5−20_: resistance at 5 Hz minus resistance at 20 Hz*.

The patient was raised in Mexico and reported no childhood history of lung problems. He denied a history before 2010 of frequent respiratory infections, exercise intolerance, frequent cough, or other breathing problems that would indicate asthma or other underlying lung disease. He immigrated to the United States in the late 1990s. He had worked at a hard metal mine in Mexico for <1 year; he had no other work history concerning for lung disease. He reported no notable travel, hobbies, or animal exposures. Pertinent negatives on review of systems included fever, chills, night sweats, weight loss, hemoptysis, and skin rashes. His physical examination findings were unremarkable; lungs were clear to auscultation bilaterally with no wheezes, crackles, or rhonchi. His oxygen saturation was 94% on room air.

**Figure 1:**
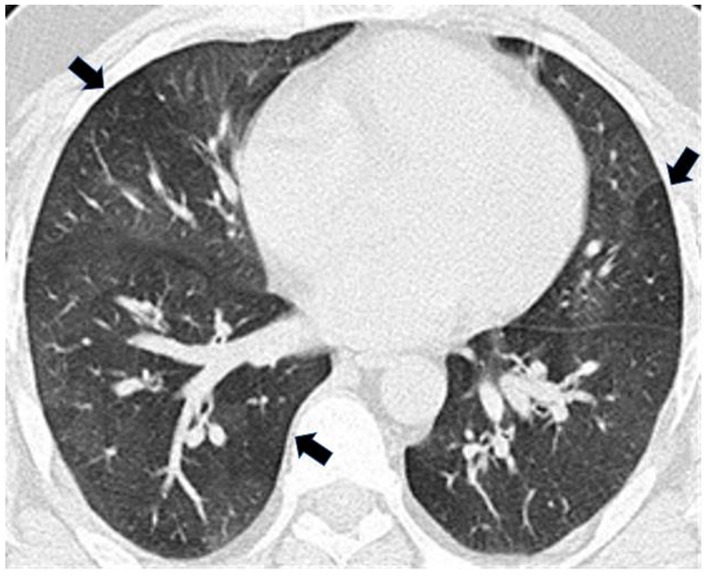
Expiratory high-resolution computed tomography (HRCT) revealed bilateral mosaic attenuation (arrows) consistent with air trapping.

**Figure 2:**
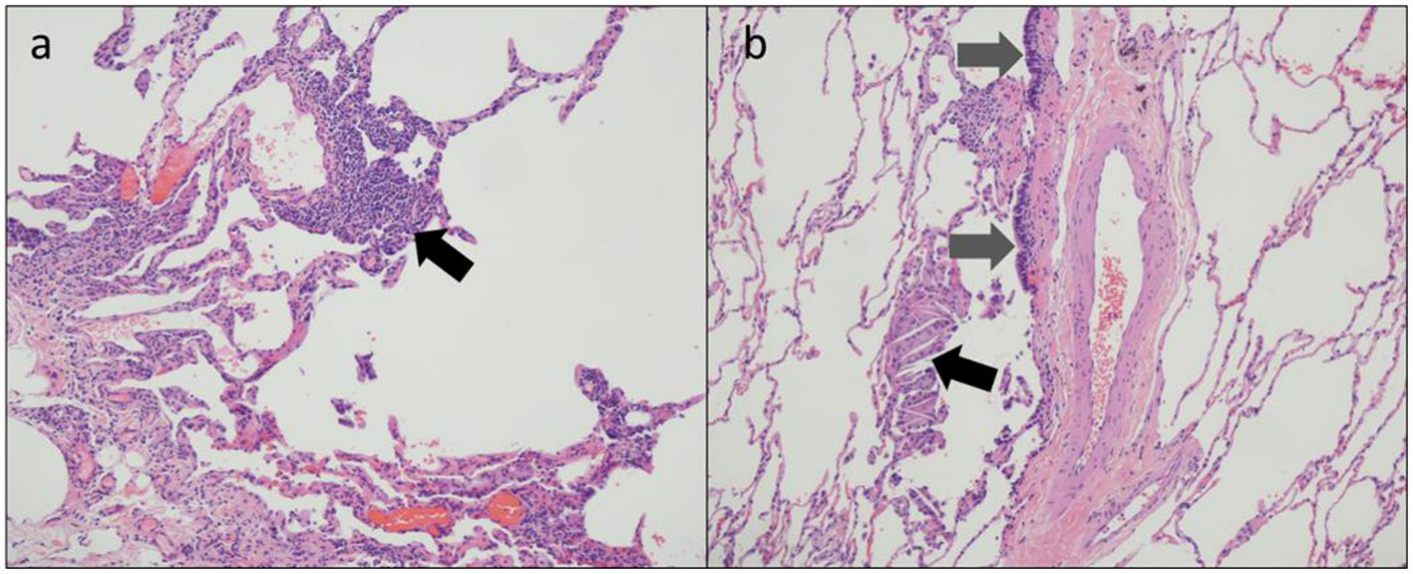
Thoracoscopic lung biopsy from a coffee roasting/packaging facility worker exposed to diacetyl showing **(a)** perivascular inflammatory infiltrates [black arrow] and **(b)** respiratory epithelium [gray arrows] and multinucleate giant cells with cholesterol clefts [black arrow].

Full pulmonary function testing confirmed a moderate mixed pattern on spirometry and demonstrated normal total lung capacity with reduced diffusing capacity (Table 1). Inspiratory and expiratory chest high-resolution computed tomography (HRCT) revealed bilateral mosaic attenuation, consistent with air trapping (Figure 1). Thoracoscopic lung biopsy revealed focal mild cellular bronchiolitis and pleuritis, including perivascular inflammatory infiltrates (Figure 2a; black arrow), and peribronchiolar giant cells/granulomas (Figure 2b; black arrow), but no evidence of fibrosis or destruction of the bronchioles. The patient was diagnosed with flavoring-related lung disease and instructed to limit his exposure to diacetyl and 2,3-pentanedione.

**Figure 3:**
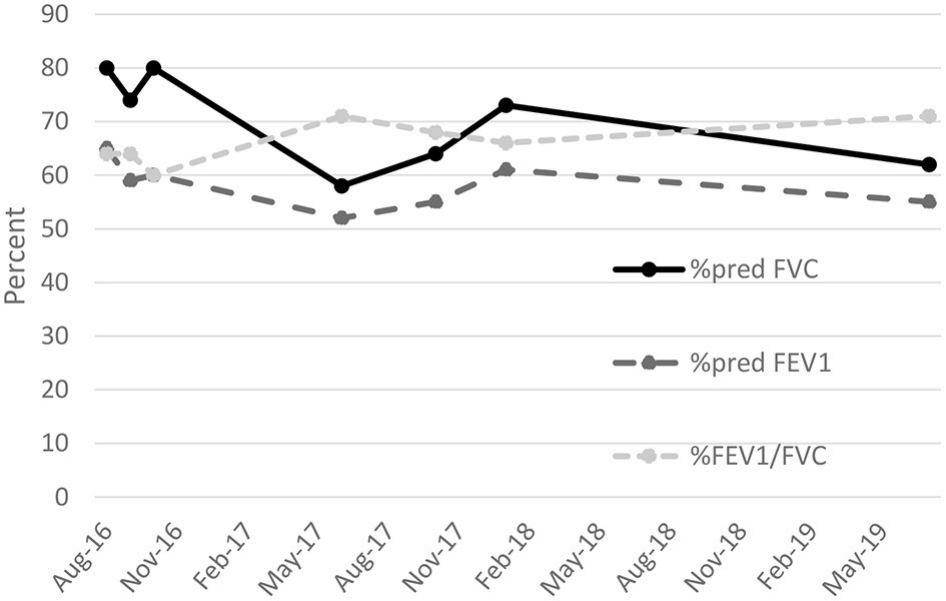
Percent predicted forced expiratory volume in 1 second (%pred FEV_1_). percent predicted forced vital capacity (%pred FVC), and ratio of FEV_1_ to FVC expressed as a percent (%FEV_1_/FVC) for patient diagnosed with flavoring-related lung disease, August 2016 to July 2019.

The patient's employer moved him from the production area of the coffee facility to an offsite warehouse. He left employment at the coffee facility in 2018. He subsequently worked for a retailer as a custodian and was advised by his pulmonologist to avoid ammonia and floor cleaners during work. He most recently worked in landscaping. He continued to experience dyspnea on exertion and a non-productive cough. He was treated with a 1-year course of azithromycin, followed by a combination of inhaled corticosteroids, beta-agonists, and anticholinergics commonly used for chronic obstructive pulmonary disease. He also received a course of oral corticosteroids for an acute exacerbation in 2018. Periodic spirometry through 2019 (Table 1 and Figure 3), demonstrated persistent mixed pattern without improvement despite cessation of exposure.

NIOSH recommended exposure limits (RELs) are 5.0 parts per billion (ppb) for diacetyl and 9.3 ppb for 2,3-pentanedione for an 8-h workday during a 40-h workweek (11). In 2016, we conducted air sampling for diacetyl and 2,3-pentanedione at the coffee roasting and packaging facility. Full-shift personal air-samples collected on other workers while duties were performed in the flavoring and grinding areas where the patient worked measured elevated levels of diacetyl (41–421 ppb) and 2,3-pentanedione (22–276 ppb) and were 2–84-fold higher than the NIOSH RELs. Short-term personal air samples collected on other workers during flavoring and grinding tasks were also high and ranged from 521 to 2,173 ppb diacetyl and 345 to 1,445 ppb 2,3-pentanedione during flavoring tasks and 47–81 ppb diacetyl and 21–50 ppb 2,3-pentanedione during grinding tasks. All 15-min samples collected while a worker flavored coffee exceeded the NIOSH short-term exposure limit of 25 ppb for diacetyl and 31 ppb for 2,3-pentanedione.

## Discussion

We describe a case of advanced lung disease in a coffee worker following prolonged exposure to high levels of diacetyl and the related chemical, 2,3-pentanedione, with no medical, other occupational, or avocational histories that could account for his illness. Additionally, noninvasive diagnostic test results including lung function testing and HRCT were consistent with previously-described cases of flavoring-related lung disease, including in coffee roasting and packaging workers. Following cessation of exposure to flavorings, his respiratory symptoms and lung function largely stabilized but did not return to normal.

It is notable that lung histopathology in this case was not consistent with obliterative bronchiolitis. Because of the variable findings of noninvasive diagnostic testing, lung biopsy is performed on some workers suspected of having flavoring-related lung disease. The diagnosis commonly associated with flavoring-related lung disease, obliterative bronchiolitis, stems from the pathologic findings from some lung biopsies in workers exposed to flavorings: destruction of the small airways marked by concentric fibrosis of the bronchioles (3). Similar to the noninvasive diagnostic testing, not all lung biopsies on exposed workers demonstrate these characteristic findings. The disease is often patchy, so the findings could be missed because of sampling error (12). The patient's tissue sample seemed to be adequate. Regardless, some evidence indicates that flavoring-related lung disease has a broader histopathologic spectrum. Lung biopsies in some workers diagnosed with flavoring-related lung disease have shown granulomatous inflammation, which is characteristic of other lung diseases including hypersensitivity pneumonitis (2, 6, 7). Pleural proliferation of mesothelial cells and eosinophils also have been observed, as have emphysematous changes and interstitial fibrosis (2, 13, 14). Thus, while this patient's lung biopsy did not demonstrate findings of obliterative bronchiolitis, a precedent exists for ascribing his histopathology to flavorings exposure, supported by his work history, symptoms, and functional and radiographic abnormalities. Furthermore, the cellular bronchiolitis identified could represent an inflammatory stage of the disease preceding development of the concentric bronchiolar narrowing and luminal obstruction classic for obliterative bronchiolitis.

This patient's functional, radiographic, and histopathologic findings could be attributable to subacute hypersensitivity pneumonitis prompted by a workplace antigen (15). Exposure to coffee dust has been associated with immune sensitization and occupational asthma (16, 17). Exposure to castor beans from cross-contamination of bags used to transport coffee are also associated with asthma in the coffee industry (18). A case of hypersensitivity pneumonitis in a coffee worker was previously reported (19), although the authors subsequently reconsidered this diagnosis when the patient developed rheumatoid arthritis and ultimately attributed his pulmonary disease to autoimmunity rather than coffee dust exposure (20). We are unable to find other reports of hypersensitivity pneumonitis in coffee workers. Notably, diacetyl and its substitutes have been found to stimulate lymphocyte proliferation in a murine model, demonstrating the potential for hypersensitivity responses (21). Thus, it is possible that exposure to flavoring chemicals for this patient caused cellular bronchiolitis and granulomatous changes via an immune-mediated mechanism rather than epithelial necrosis and airway fibrosis via disruption of protein homeostasis (22). Measurements of specific immunoglobulins or lymphocyte proliferation in response to workplace antigens were not available to help distinguish these possibilities.

Patients with flavoring-related lung disease are often diagnosed with more common obstructive lung diseases such as asthma or chronic obstructive pulmonary disease (COPD) before flavoring-related lung disease is correctly diagnosed (23). After developing dyspnea, the patient reported frequent respiratory infections and was prescribed medications for suspected pneumonia before his diagnosis of flavoring-related lung disease. In retrospect, these episodes were likely attributable to his work-related lung disease. Flavoring-related lung disease can be misdiagnosed because of its relatively rare occurrence compared with other obstructive lung diseases, but also because clinical features of the disease can vary. The most common symptoms are shortness of breath, dry cough, and chest tightness with no work-related pattern; however, upper respiratory symptoms including mucous membrane irritation and rhinosinusitis can also occur (1, 24, 25). Although fixed airflow obstruction is a common finding in flavoring-exposed workers diagnosed with clinical bronchiolitis obliterans, spirometry results vary and can include restrictive, mixed, or even normal patterns (5, 6, 25–29); the patient's most common spirometric interpretation was mixed obstructive and restrictive patterns. HRCT findings commonly demonstrate a mosaic attenuation pattern with air trapping in workers diagnosed with flavoring-related lung disease, but HRCT results are often nonspecific and can vary (30); this patient's HRCT findings were consistent with air trapping.

No other workers at the patient's coffee roasting and packaging facility were diagnosed with flavoring-related lung disease following the NIOSH medical survey. Ninety-nine (83%) of 120 employees participated in the medical survey; 15 reported grinding or flavoring tasks and these participants were nearly 4-times more likely to report chest tightness in the last 12 months (odds ratio 3.7; 95% confidence interval: 1.0–13.4) (31). Five (5%) of 98 other participants who completed spirometry at the facility also had abnormal spirometry, including three with mild restrictive patterns, one with a moderate mixed pattern, and one with mild obstruction (31). The most commonly reported symptoms by participating workers were nose and eye symptoms, reported by 46 and 43% of workers, respectively. Wheezing or whistling in the chest was the most commonly reported lower respiratory symptom (18%), followed by shortness of breath, breathing trouble, and chest tightness (17% each) (31). Some participating workers reported their symptoms were better away from work or caused or aggravated by work. Following the NIOSH health hazard evaluation, the employer instituted a medical surveillance program that included repeating spirometry every 6 months to identify employees who might be developing work-related lung disease (e.g., asthma, flavoring-related lung disease).

Other alpha-diketones, including 2,3-pentanedione and 2,3-hexanedione, have been used as substitutes for diacetyl in some industries after the association of diacetyl and flavoring-related lung disease was observed. However, evidence indicates these substitutes can result in similar pathologic findings as diacetyl and therefore are not safe alternatives (32–34). More recently, another structurally similar compound, methylglyoxal, was toxic at even lower concentrations than diacetyl in animal models (22). Headspace air sampling results from the liquid flavorings sampled at the coffee roasting and processing facility where the patient worked all contained diacetyl (up to 10,741 ppb); most contained 2,3-pentanedione (up to 6,517 ppb); none had detectable levels of 2,3-hexanedione; and we did not test for methylglyoxal (31). A recent study of headspace samples from dozens of liquid flavorings found a majority of the flavorings tested had diacetyl, 2,3-pentanedione, or both as volatile constituents in the headspace. Diacetyl and 2,3-pentanedione were not listed on the Safety Data Sheets of the flavorings tested because of trade secret designations. However, inclusion of diacetyl and 2,3-pentanedione on Safety Data Sheets is vital to protecting downstream users such as coffee roasting and processing facilities that add liquid flavorings, from unrecognized exposure and potential respiratory disease (35). Flavorings from the facility where the patient worked were tested and contained flavoring chemicals that were not disclosed on the Safety Data Sheets.

At the coffee roasting and packaging facility where the patient worked, ~12 million pounds of coffee were roasted and packaged annually as of 2016, and roughly 60% of the coffee produced was ground. The patient worked in the flavoring and grinding area full-time during 2009–2013, and then at least 20% of the time an additional 4 years. To grind coffee, an automated pneumatic system was used to pull whole beans from a storage silo into either one of two grinders. Both grinders operated on a continuous process and would grind a full silo of roasted coffee in 45–50 min. If grinding a full silo of roasted coffee, the patient would set-up the silo and grinder and then walk away to perform other tasks. Ground coffee was sent through an automated system to another silo for storage until needed for further processing (e.g., flavoring or packaging). To flavor coffee, whole bean or ground coffee was sent to the flavoring room by a pneumatic system that would pull roasted coffee from the silo into the ribbon blender in the flavoring room. The patient would mix a 40-pound pail of liquid flavorings and would manually add liquid flavorings to the ribbon blender while the blender mixed the whole beans or ground coffee with the flavorings. Liquid flavorings were manually added and mixed over a specified time. Once complete, the flavored coffee was emptied into a silo for storage until needed for packaging. The flavoring operation was isolated in a designated flavoring room prior to our investigation. Shortly before our on-site investigation in 2016, employees working in the roasting, grinding, and flavoring areas were required to wear fit-tested half-face or full-face respirators equipped with organic vapor cartridges. Following our investigation, the grinding area was also isolated. Additional engineering control solutions including enclosing and automating the flavoring system and increasing ventilation in the flavoring and grinding rooms were also implemented to reduce potential exposures for workers assigned to duties in these areas.

Our investigation was limited by several factors. While the patient had no known environmental or other occupational exposures attributable to his illness, we do not have a detailed work history and understanding of potential respiratory hazards from his previous work in Mexico before immigrating to the United States in the 1990s. Additionally, no historical air sampling results for diacetyl and 2,3-pentanedione were available from 2009 to 2013 when the patient began working in the flavoring and grinding areas and developed symptoms. Worker participation in the NIOSH health hazard evaluation was good at 83% of current workers, however, our evaluation did not include former workers who could have experienced higher historical exposures. One other current employee was reported to have long-term exposures to flavorings but did not participate in the NIOSH medical survey or respond to several attempts to make contact; it could be possible we missed other cases of flavoring-related lung disease associated with working at this coffee roasting and packaging facility. Additionally, the patient did not undergo laboratory evaluation for connective tissue diseases like rheumatoid arthritis, which can cause bronchiolitis (36). In some cases, lung disease can be the first manifestation of connective tissue disease (37), but notably, the patient did not report joint paint or other symptoms that would suggest rheumatoid arthritis or another connective tissue disease through 2019.

Despite biopsy findings not supportive of obliterative bronchiolitis, medical evaluations, including lung function testing and HRCT, air sampling data, and the lack of other explanatory medical, occupational, or avocational histories indicate this worker developed work-related, airway-centric lung disease, most likely attributable to inhalational exposure to flavorings following years of high exposures to diacetyl and 2,3-pentanedione at a coffee roasting and packaging facility. Clinicians should be aware that lung pathology could vary considerably in workers with suspected flavoring-related lung disease. Public health practitioners should be aware that workers at coffee roasting and packaging facilities, particularly those that add flavorings to coffee, could be at risk for flavoring-related lung disease. Furthermore, if a single case of flavoring-related lung disease is identified, a thorough workplace investigation is warranted to evaluate and reduce exposure to diacetyl and related compounds, as well as identify any additional cases and remove from exposure.

## Data Availability Statement

Due to restrictions imposed under the US privacy act and the limitations of what participants consented to, the data underlying the analyses presented, beyond what is provided in the paper, are confidential and not available to researchers outside the National Institute for Occupational Safety and Health (NIOSH). For more information about NIOSH's policy regarding sensitive data, see https://www.cdc.gov/niosh/ocas/datahandle.html.

## Ethics Statement

The NIOSH Institutional Review Board reviewed and approved this study (NIOSH Protocol 17-RHD-06XP). The patients/participants provided their written informed consent to participate in this study.

## Author Contributions

RH, BB, RB, JC-G, and KC contributed to the conception and presentation of the case report. EK, AR, and VR provided clinical expertise and interpretations. RH wrote the first draft of the report. All authors contributed to manuscript revision, read, and approved the submitted version.

## Conflict of Interest

The authors declare that the research was conducted in the absence of any commercial or financial relationships that could be construed as a potential conflict of interest.
